# Spatial and temporal distribution of four malaria vector species and their relative contributions to *Plasmodium falciparum* transmission along the south–north transect of Benin, West Africa

**DOI:** 10.1186/s41182-025-00822-5

**Published:** 2025-10-30

**Authors:** Serge Akpodji, Clément Agbangla, Germain Gil Padonou, Zul-Kifl Affolabi, Zinsou Côme Koukpo, Constantin Adoha, Steve Zinsou Hougbe, André Sominahouin, Filémon Tokponnon, Razaki A. Osse, Olivier Oussou, Bruno Adjottin, Esdras Mahoutin Odjo, Boulais Yovogan, Roseric Azondékon, Albert Salako, Martin Akogbéto

**Affiliations:** 1 Centre de Recherche Entomologique de Cotonou, (CREC), Cotonou, Bénin; 2 Université d’Abomey-Calavi, (UAC), Abomey-Calavi, Bénin; 3 Université Nationale d’Agriculture du Bénin, (UNA), Kétou, Bénin

**Keywords:** Malaria vector, Transmission, Sporozoite load, EIR

## Abstract

**Background:**

To help with planning malaria vector control in Benin, the National Malaria Control Program launched a study to update the distribution of major malaria vectors and their role in *Plasmodium falciparum* transmission. The study aimed to go beyond the standard entomological inoculation rate (EIR) by incorporating the average sporozoite load of the mosquitoes. This is because the parasite load is a key factor in a successful infection. The research proposed combining the average *P. falciparum* sporozoite load with EIR to better determine the vectors’ true contribution to malaria transmission.

**Methods:**

The study was conducted across 18 communes in Benin. Within each commune, two villages were chosen for mosquito collection using human landing catches (HLC) and pyrethrum spray catches (PSC). Heads and thoraxes from female *Anopheles gambiae s.l.* and *Anopheles funestus* mosquitoes were analyzed for the *Plasmodium falciparum* circumsporozoite antigen using the ELISA/CSP method. The corresponding carcasses were used for species identification via PCR. The *P. falciparum* sporozoite load was quantified in CSP ELISA-positive mosquitoes using the NZYTech real-time PCR kit. The contribution of vectors to *P. falciparum* transmission was first estimated by considering both their infection and bite rates. Subsequently, the relative contribution to transmission was further assessed by correlating the *P. falciparum* sporozoite load of the primary vectors with EIR.

**Results:**

*Anopheles coluzzii* is responsible for 63.01% of malaria transmission, with an EIR of 87.7 infecting bites per person per year. This is followed by *Anopheles gambiae,* which accounts for 36.7% of transmission and has an EIR of 51.1 infecting bites per person per year. The contribution of *Anopheles funestus* is 0.24%. The study found that *An. gambiae* carries a higher load of *Plasmodium falciparum* sporozoites than *An. coluzzii*. Specifically, approximately 30% of *An. gambiae* individuals carried more than 10,000 sporozoites in their salivary glands, while less than 10% of *An. coluzzii* individuals had a comparable load.

**Conclusion:**

This study clarifies the true contribution of malaria vectors to *Plasmodium falciparum* transmission by linking sporozoite load to the EIR. The findings will allow for a more accurate assessment of the vectors’ role in *P. falciparum* transmission in Benin.

## Background

In tropical Africa, Four anopheline species, including *Anopheles* *gambiae*, *An*. *coluzzii*, *An*. *arabiensis*, and *An*. *funestus*, are responsible for transmitting malaria to humans. [1, 2]

In Benin, studies conducted by Akogbéto et al*.* [3] have shown that there are three species of the *Anopheles gambiae* complex, including *An. gambiae* and *An. arabiensis* in different regions and *An. melas* in the southern lagoon coastal zone [4]. These studies showed that *Anopheles gambiae* was the most prevalent malaria vector with a wide distribution throughout the country. Historically, *Anopheles arabiensis*, a species typically associated with dry savannah habitats, was previously documented exclusively in Bétérou, northern Benin [3, 4]. However, recent surveys have revealed its presence in central Benin, more specifically in Glazoué and Bantè. The expansion of *Anopheles arabiensis* from the northern region to the southern region was already reported by Gnanguenon et al*.* [5]. These findings indicate that the geographical distribution of this vector is expanding, suggesting its adaptation to more diverse ecological habitats. Furthermore, the prolonged dry seasons observed in Benin in recent years, potentially attributable to climate change [6], may have contributed to the development and spread of this vector.

*Anopheles funestus* is widespread in Africa [1]. This group includes several morphologically similar species that can be distinguished at certain stages of larval development. The *Anopheles funestus* group is present in Benin, but it is less widespread and has a lower density than the major malaria vectors *An. gambiae* and *An. coluzzii*. Several studies have shown the presence of two species of the *Anopheles funestus* group: *An. funestus s.s*. and *An. leesoni.* These two species are found to be relatively predominant between the end of the rainy season and the beginning of the dry season (October and November) [7–9].

Recent localized surveys have indicated changes in the initial distribution of malaria vectors. The high presence of *Anopheles arabiensis* in Benin and the colonization of new territories by this species could be related to the rise in temperature observed in the country in recent years. Similarly, the decrease in the *Anopheles melas* population in Benin is likely linked to recent heavy rainfall and flooding in the coastal lagoon region in Benin. According to Akogbéto et al*.* [10], this flooding causes the desalination of breeding sites, which negatively impacts the species’ development.

Studies conducted in the Alibori and Donga regions of Benin have demonstrated that *Anopheles gambiae* exhibits a higher rate of anthropophily compared to *An. coluzzii*, while both species present similar sporozoite indices [11]. These investigations further identified seasonal variations in the contributions of these two species to malaria transmission. However, the scope of these studies was limited to northern Benin and did not evaluate the potential involvement of other vector species in the transmission dynamics.

The intensity of malaria transmission is quantified using the entomological inoculation rate (EIR), which measures the number of infective mosquito bites per person per unit of time. This metric assumes that all mosquitoes with sporozoites in their salivary glands have equal infectivity. However, recent studies [12, 13] have shown that mosquitoes with high parasite loads are more likely to successfully transmit the pathogen than those with low parasite loads. This suggests a heterogeneity among infected mosquitoes, where some individuals have a greater capacity for transmission than others [14, 15]. A better understanding of the variation in parasite loads among different vector species could lead to improved predictions of malaria transmission intensity in various ecological settings.

This study was conducted across 18 communes in Benin, covering a range of ecological zones along a south–north transect. The findings update the existing knowledge on the distribution of key malaria vectors. The research also links the density of each species to its *Plasmodium falciparum* infectivity, which helped to determine the seasonal and spatial contribution of each species to malaria transmission. Furthermore, the study associates the *P. falciparum* sporozoite loads in the salivary glands of each species with their respective entomological inoculation rates (EIRs), providing a deeper understanding of their roles in transmission. The results of this work will assist the National Malaria Control Program (NMCP) in tailoring its vector control strategies to the specific intensity of transmission.

## Methods

### Study site

**Fig. 1:**
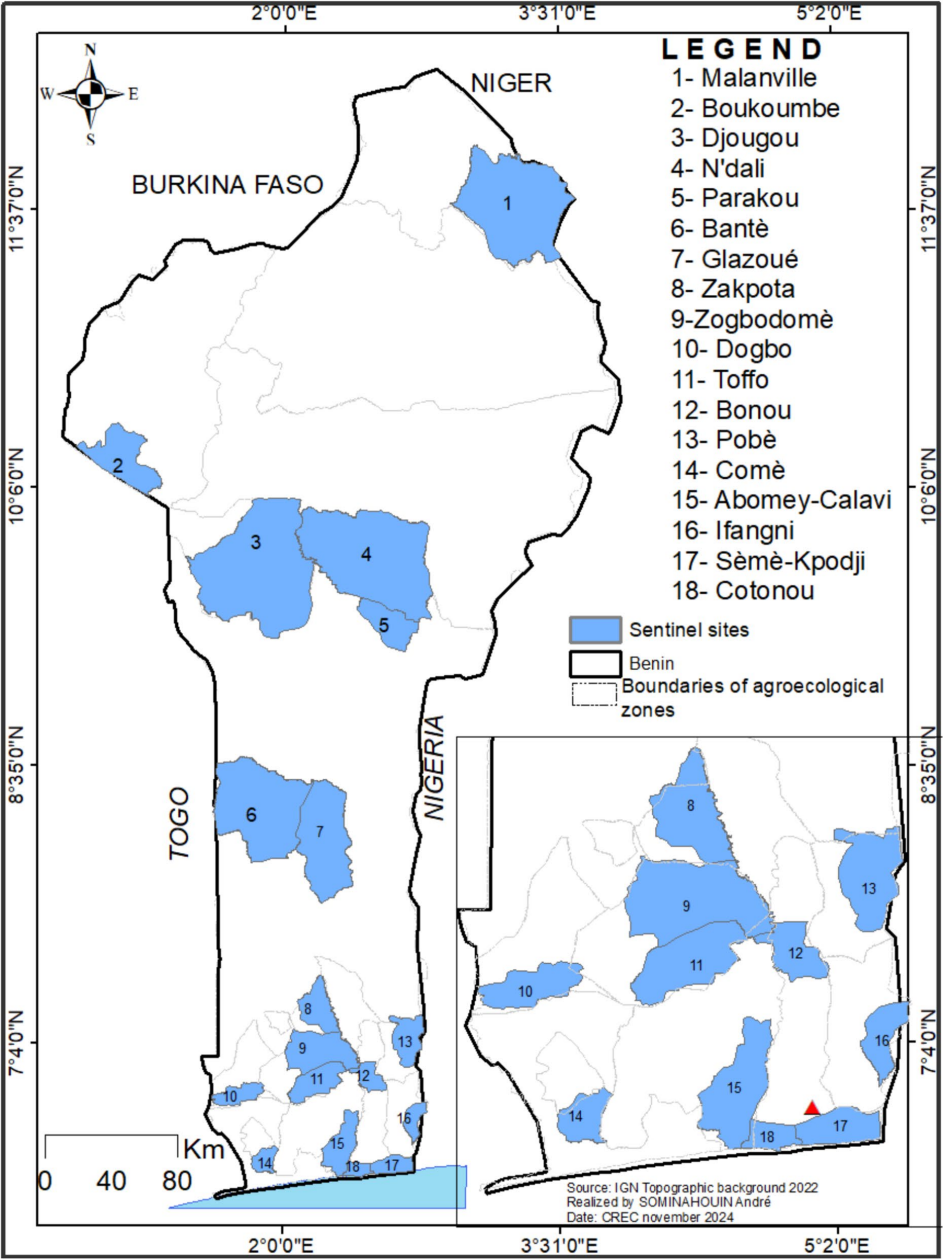
Geographical distribution of the study sites for the spatial and seasonal variation in malaria vectors (2021–2023)

This study was conducted in 18 communes in Benin, selected along a north–south transect (Fig. 1). Benin is located between the equator and the Tropic of Cancer, specifically between 6°30' and 12°30' North latitude, and 1° and 3°40' East longitude. The country covers an area of 114,763 km^2^ [16]. Benin's climate varies by region: in the south, it has a sub-equatorial climate with two rainy seasons and two dry seasons [16]. The center has a transitional Sudano-Guinean climate with four seasons, where the distinction between the two rainy seasons is less pronounced, and the north has a Sudanese climate with one wet and one dry season [17].

### Adult anopheline collection

**Fig. 2:**
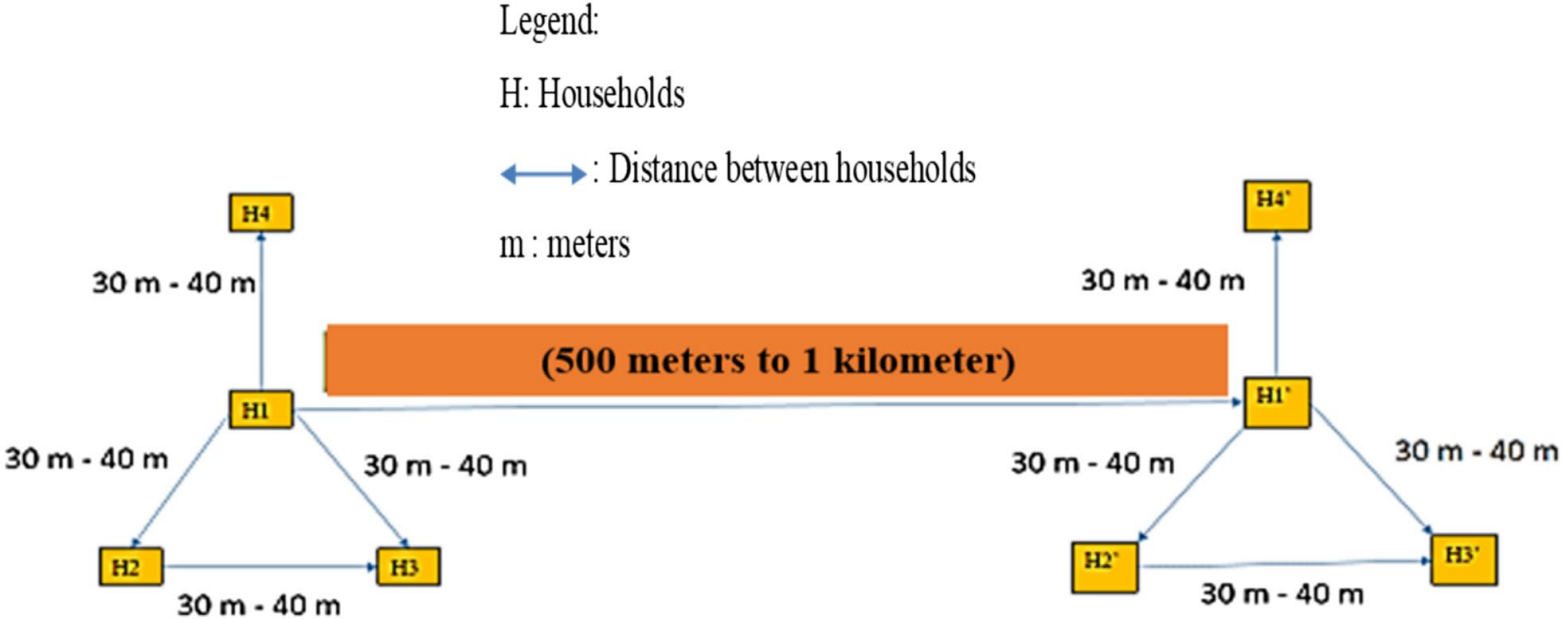
Distribution of houses to investigate for HLC

Mosquitoes were collected at each site using two methods: human landing catch (HLC) and pyrethroid spray catch (PSC). In each commune, a rural and an urban village were selected. For the HLC method, eight households (H) were selected per village—four at high altitude and four at low altitude–to better represent the diverse environmental conditions where the vectors are found. The two groups of households in each village were situated 500–1000 m apart. Within each sampling group, a reference household was randomly selected, with additional surrounding households randomly chosen at distances of 30–40 m to the north, southeast, and southwest of the reference household (Fig. 2).

Mosquitoes were collected during two periods: January to July 2021 (four rounds) and August 2022 to July 2023 (five rounds). Collections took place during both the dry season (December, January, February, and March) and the rainy season (May, July, August, and October). Within each household, mosquitoes were collected for one night from 7:00 p.m. to 7:00 a.m., both indoors and outdoors.

For pyrethrum spray catch (PSC) collections, ten additional houses per village were selected. Mosquitoes that fell to the ground 15 min after insecticide spraying were collected and placed into petri dishes. All collected malaria vectors (*Anopheles gambiae s.l*., *An. funestus* group, and *An. nili* group*)* were used for species identification and the detection of *Plasmodium falciparum* infection.

### Sample processing

The collected mosquitoes were first identified morphologically using the taxonomic keys of Gillies and De Meillon [1] and Gillies and Coetzie [18]. The *Anopheles* mosquitoes were then stored in labeled Eppendorf tubes with silica gel. A subsample of 4797 mosquitoes was processed for *Plasmodium falciparum* infection via the ELISA CSP (enzyme-linked immunosorbent assay) method on the heads and thoraxes of female *Anopheles gambiae s.l*. and *An. funestus*, as described by Wirtz et al*.* [19]. The carcasses of these mosquitoes were subsequently used for PCR-based species identification and molecular form detection, following protocols outlined in [20, 21].

### Determination of the sporozoite load of* Plasmodium falciparum *in* Anopheles gambiae s.l.*

The quantification of *Plasmodium falciparum* sporozoites using real-time PCR, which amplifies specific *Plasmodium* DNA (plasmepsin), and detects the amplified product with fluorescent markers. This method quantifies sporozoites in the sample, specifically in the heads and thoraxes of *Anopheles gambiae* and *An*. *coluzzii* that tested positive for CSP ELISA. Following DNA extraction of *Plasmodium falciparum* from CSP ELISA-positive samples, real-time PCR testing was performed using the NZYTech kit (reference MD 02411, 150 reactions). This kit is designed to provide a broad detection profile while remaining specific to the *Plasmodium falciparum* genome. The primer and probe sequences in this kit show 100% homology with a wide range of *Plasmodium falciparum* sequences, according to an exhaustive bioinformatic analysis. The reaction mixture was amplified in a thermocycler, and fluorescence was measured in real time at each cycle. The amount of amplified DNA and, therefore, the amount of sporozoites was determined by comparing the fluorescence curve with that of known controls.

### Statistical analysis

The study evaluated three key entomological parameters: the human bite rate (HBR), defined as the average number of mosquito bites per person per night; the sporozoite index (SI), which is the proportion of mosquitoes of a given species that test positive for the *Plasmodium falciparum* circumsporozoite antigen; and the entomological inoculation rate (EIR), which represents the number of infective bites received per person per night.

The HBR for *Anopheles coluzzii*, *An. gambiae*, *An. arabiensis*, and the *An. funestus* s.s. was estimated by dividing the total number of mosquitoes of each species (calculated by multiplying the total number of vectors per commune by the proportion of each species in the sampled collection) by the number of collectors per commune. The SI for each *Anopheles* species was determined by dividing the number of vectors positive for the CSP ELISA test (obtained by associating mosquitoes positive for the CSP ELISA test with their species) by the total number of each species analyzed.

The contribution of each vector species was then estimated by calculating the EIR, which was derived by multiplying the species’ HBR by its SI. Statistical analysis was performed using R statistical software, version 2.8. The Poisson confidence interval calculation method [22] was used to compare the bite rate (HBR) between vectors on the one hand and the entomological inoculation rate (EIR) between vectors on the other. The Chi-squared test for comparison of proportions was used to compare the infectivity rate. These parameters were compared between the dry season and the rainy season and between the different areas. The *Plasmodium falciparum* sporozoite loads in *Anopheles gambiae* and *An. coluzzii* were compared using the Kruskal–Wallis test for both seasonal and overall loads irrespective of season.

The mean *Plasmodium falciparum* sporozoite load per mosquito species was calculated by dividing the total number of sporozoites in that species by the number of mosquitoes analyzed.

### Ethical notice

The protocol for this study was evaluated and approved by the Centre de Recherche Entomologique de Cotonou (CREC). Measures were taken to minimize risk to the mosquito collectors. All collectors were vaccinated against yellow fever and were regularly monitored. In the event of a confirmed malaria infection, they were immediately seen and treated by a team of nurses and physicians.

## Results

### Diversity and abundance of mosquitoes

A total of 79,659 mosquitoes were collected, with 56,219 captured during the rainy season and 23,440 during the dry season (Table 1). Four mosquito genera were collected in both rainy and dry seasons; of these, *Culex* spp. contributed 50.5% of the overall collections, which is stratified into 48.3% of rainy season total collections and 55.9% of dry season total collections. *Anopheles *spp*.* contributed 33.9% of overall collections, segmented as 37.3% of total collected during the rainy season and 25.8% of dry season collections. *Aedes* spp. contributed to 2.1% of the overall total collections, which can be split as 2.0% of total collections from the rainy season and 2.4% of collections from the dry season. Lastly, *Mansonia* spp. contributed to 13.5% of the overall collections, which when split by seasons of collection, showed that it constituted 12.4% and 15.9% of samples collected during the rainy and the dry seasons, respectively.

**Table 1:** Seasonal variation in the abundance of *Anopheles* species in the 18 communes

Districts	*An. gambiae s.l.*		*An. funestus group*		Other *Anopheles *spp*.*		*Aedes *spp*.*		*Culex *spp*.*		*Mansonia *spp*.*	
	Rainy	Dry	Rainy	Dry	Rainy	Dry	Rainy	Dry	Rainy	Dry	Rainy	Dry
Abomey- Calavi	37	18	0	0	5	65	22	31	814	636	22	43
Bantè	191	29	8	0	0	0	184	58	695	170	0	1
Bonou	694	234	7	0	166	8	32	18	1452	335	4525	2436
Boukoumbé	2004	2	64	0	19	0	37	0	360	88	179	1
Comè	1185	128	5	2	7	12	56	36	1933	1289	510	305
Cotonou	249	66	0	0	1	0	219	117	5541	4817	3	7
Djougou	1373	1	5	0	2	0	80	8	729	295	7	0
Dogbo	764	78	131	8	8	0	47	15	1542	487	406	81
Glazoué	2984	2	0	0	3	0	87	33	2362	442	78	8
Ifangni	5124	3660	3	0	3	0	17	7	1523	650	198	119
Malanville	1110	124	22	7	290	3	13	5	2922	469	389	78
N'Dali	207	148	0	1	0	0	5	0	598	291	1	1
Parakou	61	4	0	0	0	0	45	2	1812	560	10	0
Pobè	40	32	2	0	0	0	78	40	591	369	77	0
Sèmè-Kpodji	427	378	0	0	8	1	26	102	1239	709	35	193
Toffo	1945	217	17	3	30	74	73	39	2351	1282	468	224
Zakpota	472	725	9	2	14	1	90	37	378	47	22	212
Zogbodomey	1254	18	7	1	5	0	1	3	311	171	62	21
Total	20121	5864	280	24	561	164	1112	551	27153	13107	6992	3730

**Fig. 3:**
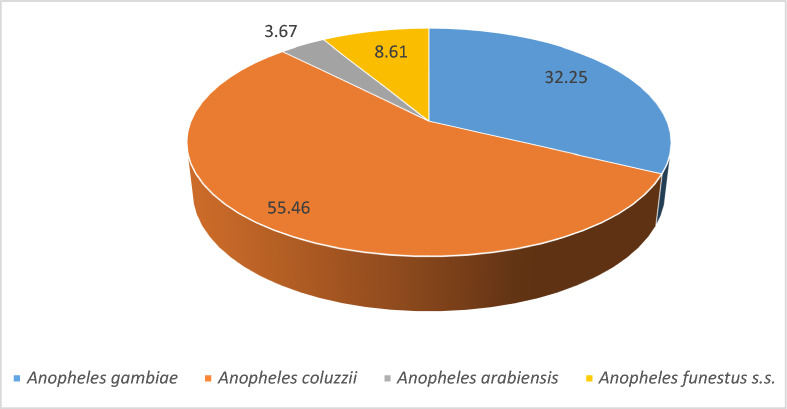
Overall variation in the relative abundance of the four malaria vectors during the rainy season

**Fig. 4:**
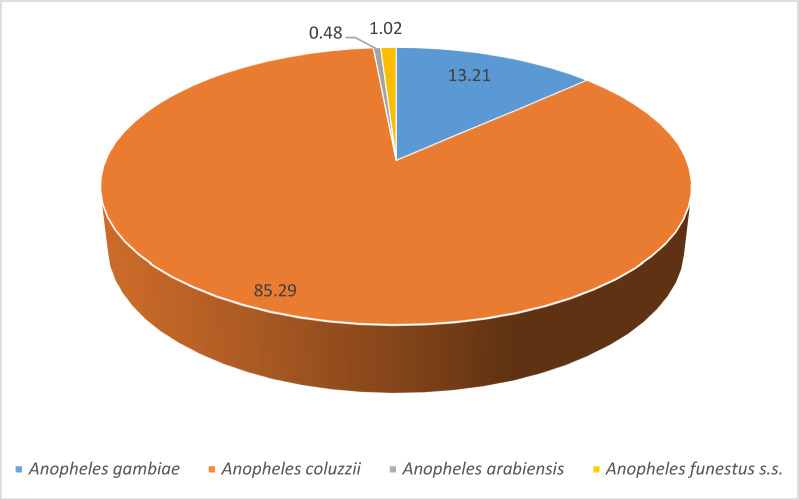
Overall variation in the relative abundance of the four malaria vectors during the dry season

**Fig. 5:**
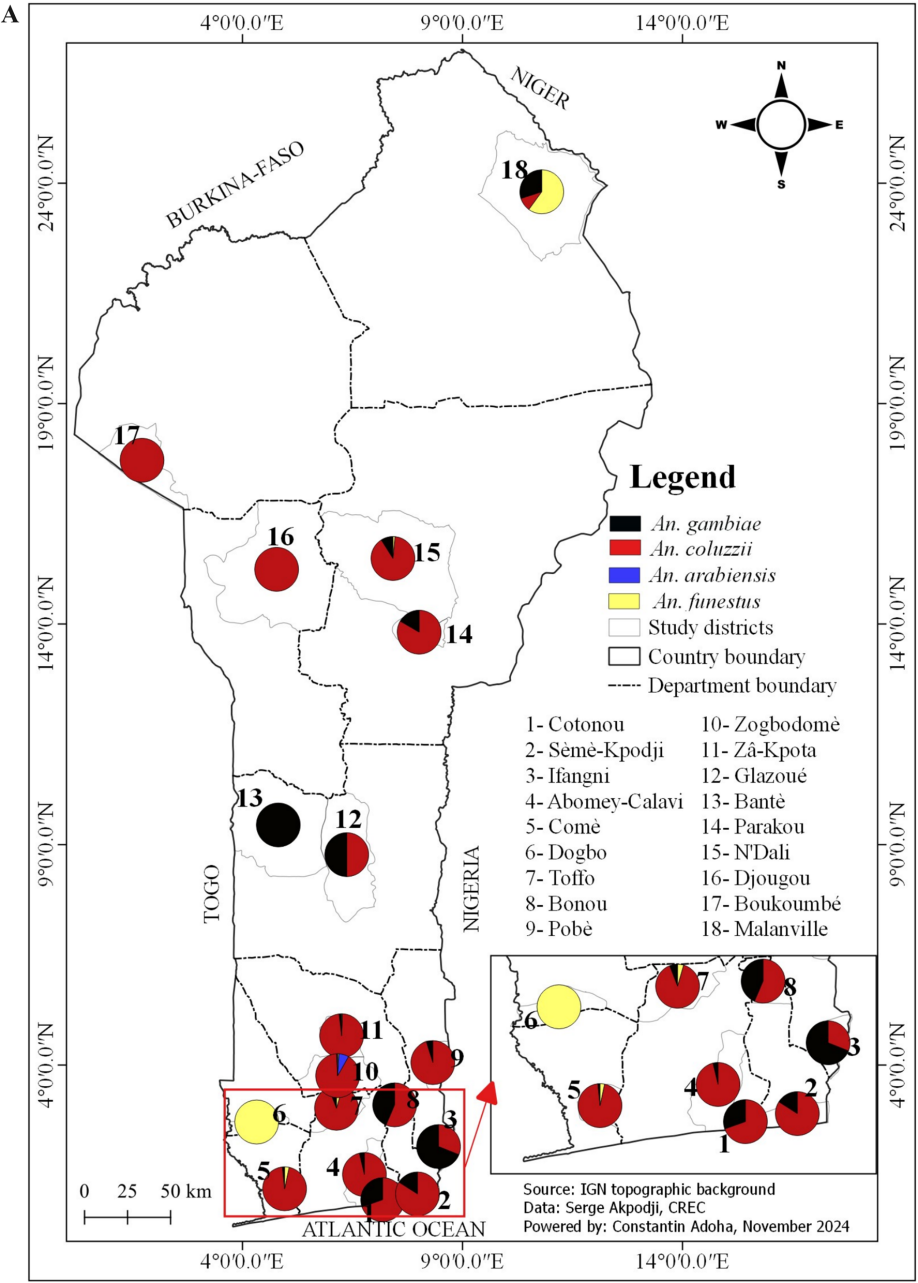

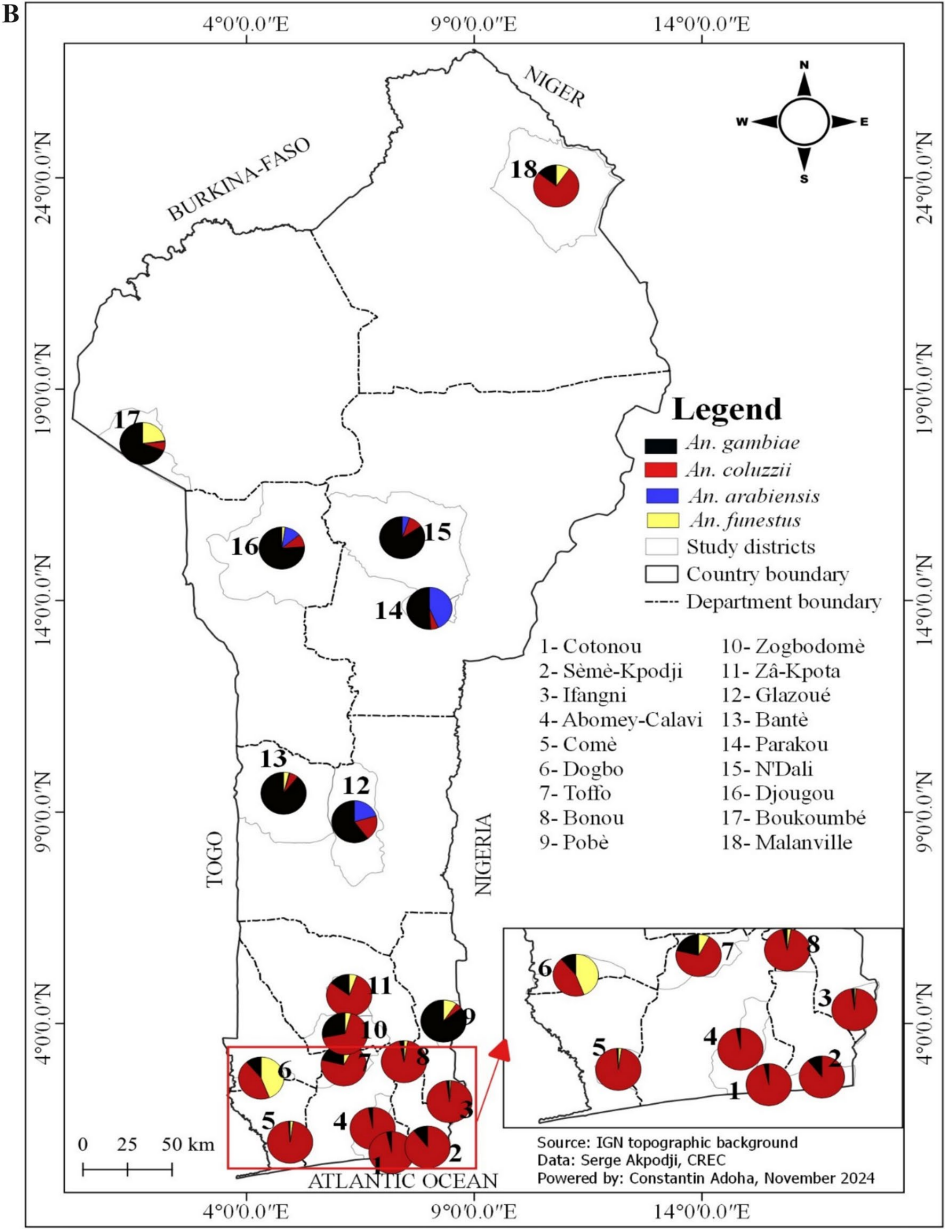
Pie chart showing the distribution of *Anopheles gambiae* and *An. coluzzii*, *An. arabiensis* and *An. funestus* during the dry season (**A**) and during the rainy season (**B**) in the 18 selected communes

Among the *Anopheles* mosquitoes*, An. gambiae s.l*. was the most prevalent species, representing 96.2% of the genus. In contrast, *Anopheles funestus group* constituted only 1.1% and was absent from many communes (Table 1). The presence of *An. funestus group* was predominantly seasonal, with 92.1% of specimens collected during the rainy season, and the highest frequency observed in the commune of Dogbo (Figs. 3, 4 and 5). *Anopheles gambiae s.l.* was found in all communes, with the largest number collected in Ifangni (Table 1).

**Fig. 6:**
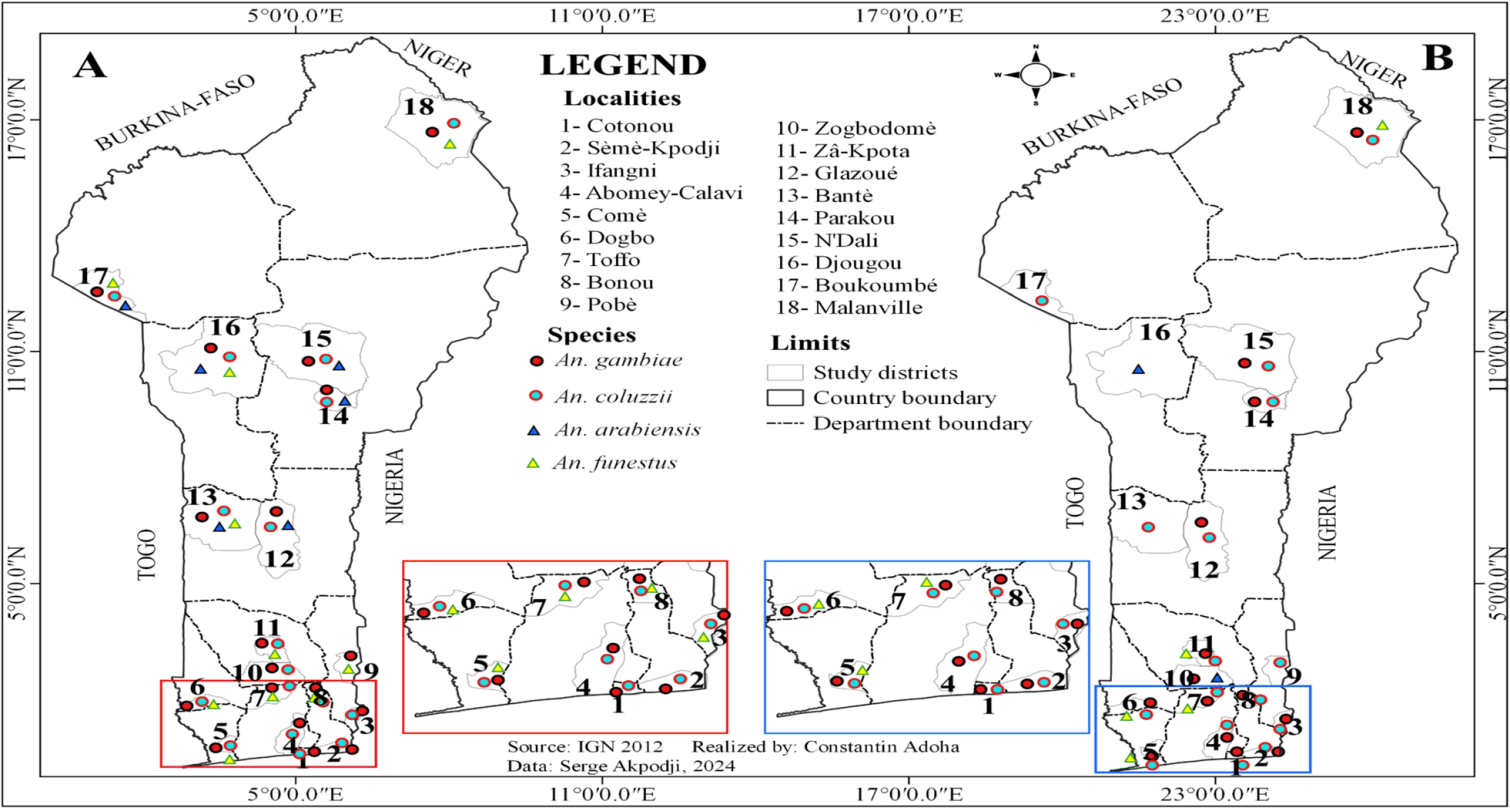
Distribution map of the 4 malaria vectors in Benin (*Anopheles gambiae*, *Anopheles coluzzii*, *Anopheles arabiensis*, and *Anopheles funestus s.s.*) in the rainy (left **A**) and dry (right **B**) season

Three species from the *Anopheles gambiae* complex were detected across all seasons: *An. gambiae*, *An. coluzzii*, and *An. arabiensis*. Overall, *Anopheles coluzzii* was the most abundant species (71.4%), followed by *An. gambiae* (25.8%) and *An. arabiensis* (2.5%). *Anopheles coluzzii* was the dominant species in the dry season, regardless of the communes, except in Glazoué, Bantè, Zogbodomey, and Parakou, where *An. gambiae* was more abundant (68% of the total collected). During the rainy season, in the areas located in the north and center of the country (N'Dali, Djougou, Boukoumbé, Bantè, Glazoue), *Anopheles gambiae* was the most represented species, except in Malanville (extreme north zone of Benin), where *An. coluzzii* was the most dominant species. In southern Benin (Cotonou, Ifangni, Bonou, Zakpota, Toffo, Zogbodomey, Abomey-Calavi, Sèmè-Kpodji, Dogbo, Comè), *Anopheles coluzzii* was the most dominant species. As for *Anopheles arabiensis*, it was only found in Zogbodomey (7.5%), N'Dali (5.8%), Djougou (17.5%), Boukoumbé (1.7%), Glazoue (27.5%), Bantè (0.8%), and Parakou (39.2%) (Table 2 and Figs. 4, 5 and 6).

**Table 2:** Seasonal variation in the relative abundance of members of the *Anopheles gambiae* complex in the 18 communes

Rainy season						Dry season				
Locations	Number tested	*Anopheles gambiae*	*Anopheles coluzzii*	Hybrids	*Anopheles arabiensis*	Number tested	*Anopheles gambiae*	*Anopheles coluzzii*	Hybrids	*Anopheles arabiensis*
		*N* (%, CI)	*N* (%, CI)	*N* (%, CI)	*N* (%, CI)		*N* (%, CI)	*N* (%, CI)	*N* (%, CI)	*N* (%, CI)
Malanville	195	31 (15.90 ± 5.13)	164 (84.10 ± 5.13)	0	0	80	3 (3.75 ± 3.34)	77 (96.25 ± 4.16)	0	0
Zogbodomey	159	46 (22.91 ± 1.03)	111 (69.81 ± 7.13)	2 (1.26 ± 0.79)	0	18	9 (50 ± 23.1)	0	0	9 (50 ± 23.1)
Toffo	179	41 (22.90 ± 6.15)	137 (76.54 ± 6.21)	1 (0.56 ± 0.03)	0	101	44 (43.56 ± 9.67)	57(56.43 ± 9.66)	0	0
Pobè	19	18 (94.74 ± 10.04)	1 (5.26 ± 0.48)	0	0	32	0	32	0	0
Bonou	180	6 (3.33 ± 2.62)	174 (96.67 ± 2.63)	0	0	58	1 (1.72 ± 0.09)	57(98.27 ± 3.34)	0	0
N'Dali	137	114 (83.21 ± 6.26)	14 (10.22 ± 5.07)	2 (1.46 ± 0.91)	7 (5.11 ± 3.69)	148	45 (30.40 ± 7.41)	103 (69.59 ± 7.41)	0	0
Boukoumbé	204	183 (89.71 ± 4.17)	18 (8.82 ± 3.89)	1 (0.49 ± 0.02)	2 (0.98 ± 0.61)	2	0	2	0	0
Djougou	167	130 (77.84 ± 6.29	17 (10.18 ± 4.59)	0	20 (11.98 ± 4.93)	1	0	0	0	1
Glazoué	161	97 (60.25 ± 7.56)	31 (19.25 ± 6.09)	0	33 (20.50 ± 6.24)	2	1 (50 ± 19.3)	1 (50 ± 19.3)	0	0
Bantè	171	159 (92.98 ± 3.83)	11 (6.43 ± 3.67)	0	1 (0.58 ± 0.02)	29	20 (68.96 ± 16.82)	9 (31.03 ± 16.83)	0	0
Parakou	109	55 (50.46 ± 9.39)	6 (5.50 ± 4.28)	1 (0.92 ± 0.05)	47 (43.12 ± 9.30)	4	3 (75 ± 42.44)	1 (25 ± 7.56)	0	0
Dogbo	155	31 (20 ± 6.30)	124 (80 ± 6.30)	0	0	68	6 (8.82 ± 6.74)	60 (88.23 ± 7.65)	2 (2.94 ± 1.87)	0
Abomey-Calavi	33	1 (3.03 ± 0.21)	32 (96.97 ± 5.85)	0	0	18	3 (16.66 ± 16.11)	15 (83.33 ± 17.21)	0	0
Ifangni	250	5 (2 ± 1.74)	245 (98 ± 1.74)	0	0	208	10 (4.80 ± 2.9)	198 (95.19 ± 2.91)	0	0
Zakpota	136	21 (15.44 ± 6.07)	113 (83.09 ± 6.30)	2 (1.47 ± 0.92)	0	725	116 (16 ± 2.67)	609 (84 ± 2.67)	0	0
Cotonou	112	4 (3.57 ± 3.44)	108 (96.43 ± 3.44)	0	0	66	4 (6.06 ± 5.76)	61 (92.42 ± 6.38	1 (1.51 ± 0.08)	0
Sèmè kPodji	207	22 (10.63±4.20)	185 (89.37±4.2)	0	0	378	7 (1.85±1.36)	371 (98.14±1.35)	o	o
Comè	173	2 (1.16±0.44)	170 (98.27±1.95)	1 (0.58±0.0 3)	0	112	1 (0.89±0.04)	110 (98.21±2.45)	1 (0.89±0.0 4)	0
Total	2747	966 (35.16)	1661 (60.46)	10 (0.36)	110 (4.00)	2050	273 (13.32)	1763 (86.00)	4 (0.20)	10 (0.49)

A total of 304 specimens from the *Anopheles funestus* group were analyzed to species level (Table 3 and Figs. 4, 5 and 6): 280 from the rainy season and 24 from the dry season, including *An. funestus s.s*. (91.8%) and *An. leesoni* (8.2%).

**Table 3:** Seasonal variation in the relative abundance of members of the *Anopheles funestus* group in the 18 communes

Rainy season						Dry season				
Locations	Number tested	*Anopheles funestus s.s.*		*Anopheles leesoni*		Number tested	*Anopheles funestus s.s.*		*Anopheles leesoni*	
		*N* (%)	IC	*N* (%)	IC		*N* (%)	IC	*N* (%)	IC
Malanville	22	21 (95.45)	77.2–99.9	1 (4.55)	0.1–22.8	7	6 (85.71)	42.1–99.6	1 (14.29)	0.4–57.9
Zogbodomey	7	7 (100)	59–100	0 (0)	0–41	1	1 (100)	2.5–100	0 (0)	0–97.5
Toffo	17	15 (88.24)	63.6–98.5	2 (11.76)	1.5–36.4	3	3 (100)	29.2–100	0 (0)	0–70.8
Pobè	2	2 (100)	15.8–100	0 (0)	0–84.2	0	0 (NA)	–	0 (NA)	–
Bonou	7	5 (71.43)	29–96.3	2 (28.57)	3.7–71	0	0 (NA)	–	0 (NA)	–
N'Dali	0	0 (NA)		0 (NA)		1	1 (100)	2.5–100	0 (0)	0–97.5
Boukoumbé	64	60 (93.75)	84.8–98.3	4 (6.25)	1.7–15.2	0	0 (NA)	–	0 (NA)	–
Djougou	5	4 (80)	28.4–99.5	1 (20)	0.5–71.6	0	0 (NA)	–	0 (NA)	–
Glazoué	0	0 (NA)		0 (NA)		0	0 (NA)	–	0 (NA)	–
Bantè	8	7 (87.5)	47.3–99.7	1 (12.5)	0.3–52.7	0	0 (NA)	–	0 (NA)	–
Parakou	0	0 (NA)		0 (NA)		0	0 (NA)	–	0 (NA)	–
Dogbo	131	122 (93.13)	87.4–96.8	9 (6.87)	3.2–12.6	8	6 (75)	34.9–96.8	2 (25)	3.2–65.1
Abomey-Calavi	0	0 (NA)		0 (NA)		0	0 (NA)	–	0 (NA)	–
Ifangni	3	3 (100)	29.2–100	0 (0)		0	0 (NA)	–	0 (NA)	–
Zakpota	9	8 (88.89)	51.8–99.7	1 (11,11)	0.3–48.2	2	2 (100)	15.8–100	0 (0)	0–84.2
Cotonou	0	0 (NA)		0 (NA)		0	0 (NA)	–	0 (NA)	–
Sèmè-Kpodji	0	0 (NA)		0 NA)		0	0 (NA)	–	0 (NA)	–
Comè	5	4 (80)	28.4–99.5	1 (20)	0.5–71.6	2	2 (100)	15.8–100	0 (0)	0–84.2
Total	280	258 (92.14)		22 (7.86)		24	21 (87.50)		3 (12.50)	

### Contribution of the different species of the *Anopheles gambiae* complex and *Anopheles funestus* group to the transmission of *Plasmodium falciparum*

Among the 4,797 *Anopheles gambiae s.l.* mosquitoes analyzed for the *Plasmodium falciparum* circumsporozoite antigen, the overall sporozoite index (SI) for the 18 communes is 4.84% in *Anopheles gambiae* and 3.13% in *An. coluzzii*. Only two specimens of *An. funestus s.s.* were positive, yielding an SI of 0.72%.

The SI varied from 0.37% in the dry season to 6.11% in the rainy season. The same trend was observed in *Anopheles coluzzii* with an infectivity of 1.70% in the dry season and 4.64% in the rainy season and in *Anopheles funestus s.s.* with an infectivity of 0.78% in the rainy season versus 0% in the dry season. In general, the sporozoite index for the three species during the rainy season was significantly higher than that observed during the dry season (*p* < 0.05) (Table 4). On the other hand, the comparison of the sporozoite index (SI) of *Anopheles gambiae* and *An. coluzzii* in the dry season and rainy season showed no significant difference (*p* ˃ 0.05), while the SI of *An. funestus* was significantly lower than the other two species in the rainy season (*p* < 0.05).

**Table 4:** Contribution of *Anopheles gambiae*, *An. coluzzii*, and *An. funestus s.s. to Plasmodium falciparum* transmission according to the season and global contribution

Season	Species	Number tested	Positive numbers	Sporozoite rate (%)	HBR/night	EIR/night	EIR/month	EIR/year	% contribution
Rainy	*An. coluzzii*	1661	77	4.64	8.74	0.410	12.30		56.87
	*An. gambiae*	966	59	6.11	5.08	0.310	9.30		43.00
	*An. funestus s.s. *	258	2	0.78	0.18	0.001	0.03		0.13
	*An. arabiensis*	110	0	0.00	0.58	0.000	0.00		0.00
	All vectors	2995	138	4.61	14.58	0.670	21.63		43.13
Dry	*An. coluzzii*	1763	30	1.70	5.08	0.090	2.59		96.64
	*An. gambiae*	273	1	0.37	0.79	0.003	0.09		3.36
	*An. funestus s.s. *	21	0	0.00	0.02	0.000	0.00		0.00
	*An. arabiensis*	10	0	0.00	0.03	0.000	0.00		0.00
	All vectors	2067	31	1.50	5.92	0.090	2.68		3.36
Overall	*An. coluzzii*	3424	107	3.13	7.78	0.240	7.31	87,600	63.01
	*An. gambiae*	1239	60	4.84	2.82	0.140	4.10	51,100	36.75
	*An. funestus s.s. *	279	2	0.72	0.12	0.001	0.03	0.330	0.24
	*An. arabiensis*	120	0	0.00	0.27	0.000	0.00	0.000	0.00
	All vectors	5062	169	3.34	10.99	0.381	11.43	139,030	36.99

The contribution to *Plasmodium falciparum* transmission by the three species (*Anopheles gambiae*, *An. coluzzii*, and *An. funestus s.s.*) was estimated from the entomological inoculation rate (EIR). Overall, the EIR of *Anopheles coluzzii* was significantly higher (87.6 infective bites per person per year) than that of *Anopheles gambiae* (51.1 infective bites per person per year) and *Anopheles funestus s.s*. (0.33 infective bites per person per year) (Table 4). As a result, *Anopheles coluzzii* accounted for 63.01% of the total transmission, followed by *Anopheles gambiae* at 36.75% and *Anopheles funestus s.s.* at 0.24% (Table 4).

In both the rainy and dry seasons, the HBR of *Anopheles coluzzii* was significantly higher than that of *An. gambiae* and *An. funestus s.s.* (*p* < 0.05). Consequently, the EIR was significantly higher in *Anopheles coluzzii* (12.30 and 2.59 infective bites per man per month) than in *An. gambiae* (9.30 and 0.09 infective bites per man per month) and in *An. funestus s.s.* (0.03 and 0 infective bites per man per month) in the rainy and dry seasons, respectively. These results indicate a transmission contribution by *Anopheles coluzzii* of 56.87% in the rainy season and 96.64% in the dry season compared to 43% and 3.36% contribution by *An. gambiae*. The transmission contribution by *Anopheles funestus s.s*. was much lower: 0.14% and 0% in the rainy and dry seasons, respectively (Table 4).

*Anopheles arabiensis* samples examined were all negative for *P. falciparum*, indicating this species was not involved in *P. falciparum* transmission during the study.

### Determination of the sporozoite load of *Plasmodium falciparum* in *Anopheles gambiae* and *Anopheles coluzzii*

The *average Plasmodium falciparum* sporozoite loads of *Anopheles gambiae* and *An. coluzzii* are summarized in Table 5. During the rainy season, the sporozoite load was significantly higher in *An. gambiae* (8,422 sporozoites) compared to *An. coluzzii* (8,153 sporozoites). When data from all seasons were combined, the difference remained significant (*p* < 0.05), with *An. gambiae* carrying an average of 8,422 sporozoites and *An. coluzzii* with 7,739. A seasonal comparison could not be made for the dry season because only one *An. gambiae* mosquito was positive for the CSP antigen, out of 31 positive mosquitoes (Table 5). Table 6 shows the percentage of individuals of the species *Anopheles coluzzii* and *Anopheles gambiae* having more or less than 10,000 *Plasmodium falciparum* sporozoites in the salivary glands stratified by season. A higher percentage of *An. gambiae* individuals (over 30%) had a load of more than 10,000 sporozoites, while less than 10% of *An. coluzzii* individuals carried a similar load (Table 6).

**Table 5:** Variation in the quantity of *Plasmodium falciparum* sporozoites in the heads and thoraxes of *Anopheles gambiae* and *Anopheles coluzzii* according to the seasons

Seasons	Species	Mean sporozoite load of *P. falciparum*	Chi-squared	df	*p*-value (Kruskal–Wallis)
Dry season	*An. coluzzii*	6083	–	–	–
	*An. gambiae*	–			
Rainy season	*An. coluzzii*	8153	15,606	1	7.8e-05
	*An. gambiae*	8422			
All seasons	*An. coluzzii*	7739	15,734	1	7.29e-05
	*An. gambiae*	8422

**Table 6:** Percentage of mosquitoes with fewer than or more than 10,000 *Plasmodium falciparum sporozoites* in the heads and thoraxes of *Anopheles gambiae* and *Anopheles coluzzii* categorized by seasons

Seasons	Species	Percentage of mosquitoes with fewer than 10,000 sporozoites in the salivary glands	Percentage of mosquitoes with more than 10,000 sporozoites in the salivary glands	Total
Dry season	*Anopheles coluzzii*	92.30% (*n* = 24)	7.69% (*n* = 2)	100% (*n* = 26)
	*Anopheles gambiae*	0% (*n* = 0)	100% (*n* = 1)	100% (*n* = 1)
Rainy season	*Anopheles coluzzii*	91.55% (*n* = 65)	8.45% (*n* = 6)	100% (*n* = 71)
	*Anopheles gambiae*	69.09% (*n* = 38)	30.91% (*n* = 17)	100 (*n* = 55)
All seasons	*Anopheles coluzzii*	90.72% (*n* = 88)	9.28% (*n* = 9)	100% (*n* = 97)
	*Anopheles gambiae*	66.07% (*n* = 37)	33.93% (*n* = 19)	100% (*n* = 56)

## Discussion

Updating the spatial and temporal distribution of the main malaria vectors and their contribution to *Plasmodium falciparum* transmission in Benin is essential for effective vector control planning and implementation. The presence of both the *Anopheles gambiae* complex and the *An. funestus* group along the north–south transect of Benin aligns with previous studies conducted in West Africa, particularly those conducted in Benin [5, 23]. The high abundance of *Anopheles gambiae s.l.* is consistent with previous results [24, 25]. The difference in frequency between *Anopheles gambiae s.l*. and the *An. funestus* is attributed to their different ecological requirements. *Anopheles gambiae s.l.* generally breeds in very transient habitats such as shallow, sunlit freshwater pools or artificial habitats [24], although they can also be common in rice paddies [25, 26]. In contrast, *Anopheles funestus* breeds mainly in marshes and other types of sheltered habitats containing vegetation which are relatively few in number in the different study areas.

In sub-Saharan Africa, several species of the *Anopheles gambiae* complex have been described: *An*. *gambiae*, *An. coluzzii*, *An. arabiensis*, *An. melas*, and *An. merus* [10, 27, 28]*.* In this study, only three species were found in the different communes, including *Anopheles gambiae, An. coluzzii*, and *An. arabiensis.* The first two were the most abundant species, representing more than 91% of the three species encountered. Their distribution follows their ecological characteristics. The abundance of *Anopheles coluzzii* in the southern communes is attributed to the abundance and permanence of water even during the dry season. This region is characterized by two rainy seasons per year, unlike the northern region, which only has one [16]. Additionally, runoff from the northern region flows south during the rainy season, causing flooding every year. These floods are also caused by a shallow water table, as well as by lakes and lagoons that release excess water onto the mainland during heavy rainfall. The favorable ecology of *Anopheles coluzzii* in this environment explains its high frequency in Cotonou (94.94%), Sèmè-Kpodji (95.04%), Abomey-Calavi (92.16%), Bonou (97.06%), Comè (98.25%), and Ifangni (96.72%). The same environment explains the high abundance of *Anopheles coluzzii* in Malanville, a commune in northern Benin. In Malanville, *Anopheles coluzzii* accounted for 96.25% of the mosquito population in the dry season and 84.10% in the rainy season, unlike other communes in the region where *An. gambiae* were more prevalent*.* Malanville is a large rice-growing area with two harvesting seasons per year, made possible by irrigation due to its proximity to the Niger River.

*Anopheles arabiensis* is typically found in dry savannahs and was previously limited to Bétérou in northern Benin [5], this study found it in Glazoué and Bantè in the central part of the country. This suggests an expansion of the vector’s geographic range and its potential adaptation to more humid environments. The spread of *Anopheles arabiensis* from the northern region to southern regions has been reported previously [5]. In addition, the long dry seasons observed in Benin in recent years due to climate change [6] may have played a role in the development of this vector.

Mosquito collections carried out in coastal lagoon environments (Cotonou, Sèmè-Kpodji) did not detect *Anopheles melas.* It is likely that this species is disappearing from the coast of Benin, probably due to urbanization and severe flooding in recent years. According to Akogbeto et al*.* [3], the development of coastal cities and flooding disrupt the ecology of *Anopheles melas* and may have led to its disappearance.

The transmission of *Plasmodium falciparum* was estimated using the sporozoite index (SI), which reflects the mosquito's capacity to develop the sporogonic cycle, and the human biting rate (HBR). The present study found that *Anopheles gambiae* and *An*. *coluzzii* had a sporozoitic index more than five times higher than that of *An*. *funestus.* The same observation was previously made by Aikpon et al. [29] in Benin, in the Atacora region. Their findings did not report a significant difference between the infection rate in *Anopheles gambiae* and *An. coluzzii*, but the rate was three times lower in *An. funestus* than it is in *An. gambiae s.l.*. *Anopheles coluzzii* is the major vector in the 18 communes due to its high frequency, especially in the south and far north. It is the main contributor to malaria transmission with an EIR of 87.7 infectious bites per person per year, with 63.01% of the transmission. The contribution of *Anopheles gambiae* was significantly lower than that of *An. coluzzii,* at 36.7% (*p* < 0.005) with an EIR of 51.1 infectious bites per person per year. Together, *Anopheles coluzzii* and *An. gambiae* are responsible for 99.7% of malaria transmission in the 18 communes, while *An. funestus* contributes a mere 0.24%*.* The involvement of *Anopheles arabiensis* in malaria transmission could not be confirmed, as all 120 specimens analyzed tested negative for *Plasmodium falciparum* sporozoites. However, its contribution is likely minimal due to its low population frequency (120 specimens out of more than 20,000 malaria vectors).

By correlating the *Plasmodium falciparum* sporozoite load with the EIR, the study determined the actual contribution of *Anopheles gambiae* and *An. coluzzii* to malaria transmission. Not all infected mosquitoes have the same probability of transmitting malaria parasites (sporozoites) and triggering malaria infection in a vertebrate host [30–32]. Previous studies [12, 13] have demonstrated that mosquitoes with a parasite load greater than 10,000 sporozoites in their salivary glands are highly likely to cause infection. This suggests that parasite load is a key factor in the success of transmission. The current study found that *Anopheles gambiae* carries more *Plasmodium falciparum* sporozoites in its salivary glands (8422 sporozoites on average during the rainy season and independently of seasons) than *An. coluzzii* (8153 sporozoites on average during the rainy season and 7739 independently of seasons). We were unable to obtain enough positive samples of *Anopheles gambiae* from the dry season, which prevented us from making a comparison. Taking the sporozoite load into account, the degree of receptivity to *Plasmodium falciparum* in *Anopheles gambiae* is significantly higher than in *An. coluzzii,* which is a different conclusion from what would be drawn by comparing their infectivity alone. Furthermore, over 30% of *Anopheles gambiae* individuals had a parasite load exceeding 10,000 sporozoites in their salivary glands whereas less than 10% of *An. coluzzii* did. These small subsets of heavily infected mosquitoes could contribute disproportionately to malaria transmission [13]. Therefore, the contribution of *Anopheles gambiae* to *Plasmodium falciparum* transmission may be higher than previously thought due to its greater capacity to promote sporogonic development and carry a higher load of *P. falciparum* sporozoites, despite its low density.

## Conclusion

Four malaria vectors are present in the 18 communes: *Anopheles coluzzii*, *An. gambiae*, *An. funestus*, and *An. arabiensis*. Of these, *Anopheles coluzzii* is the most dominant species. It is present in all areas sampled and was the only species present in certain communes. It is widespread in southern Benin and in the far north (Malanville). Its role in malaria transmission is attributed more to its relative abundance than to its vector competence. In contrast, the role of *Anopheles gambiae* in malaria transmission could be linked to its vector competence. The contribution of *Anopheles funestus* and *An. arabiensis* to malaria transmission appears to be negligible, as their distribution is localized to a few regions of Benin with very low frequencies. In conclusion, our findings highlight the importance of both *An*opheles *coluzzii* and *An. gambiae* in malaria transmission in Benin and identify key areas for vector control interventions to reduce malaria transmission.

## Data Availability

No datasets were generated or analysed during the current study.

## Abbreviations

NMCP: National Malaria Control Program
EIR: Inoculation entomological rate
WHO: World Health Organization
